# Supplementary material for: A bibliometric analysis in gene research of myocardial infarction from 2001 to 2015
Source: PeerJ. 2018 Feb 12;6:e4354. doi: 10.7717/peerj.4354 (PMC5813587; doi:10.7717/peerj.4354)
Supplement: Table S1 [file peerj-06-4354-s001.docx]

**Supplementary Table 1 Search queries used in Web of Science Core Collection**

| Set | Search Term | Results |
| --- | --- | --- |
| # 1  # 2  # 3  # 4  # 5  # 6  # 7  # 8  # 9  # 10 | TITLE: (Mycardial infarct*)  TITLE: (Non-ST Elevation Myocardial Infarct*)  TITLE: (ST Elevation Myocardial Infarct*)  TITLE: (NSTEMI)  TITLE: (STEMI)  TITLE: (MI) AND TOPIC: (Myocardial Infarction)  TITLE: (AMI) AND TOPIC: (Myocardial Infarction)  TOPIC: (gene)  # 1 OR # 2 OR # 3 OR # 4 OR # 5 OR # 6 OR # 7 (DOCUMENT TYPES: (ARTICLE OR REVIEW))  #9 AND #8 | 47325  1319  8397  303  2105  522  734  1601940  23291  1853 |

^a^ Indexes=SCI-EXPANDED, SSCI, A&HCI, CPCI-S, CPCI-SSH, ESCI. ^b^ Timespan=2001-2015.
