# Supplementary material for: A bibliometric analysis in gene research of myocardial infarction from 2001 to 2015
Source: PeerJ. 2018 Feb 12;6:e4354. doi: 10.7717/peerj.4354 (PMC5813587; doi:10.7717/peerj.4354)
Supplement: Table S2 [file peerj-06-4354-s002.docx]

**Supplementary Table 2. Number and citation frequency of published articles on the gene research of myocardial infarction indexed in the Web of Science during 2001–2015**

| Year | Counts | Citation | Average citation frequency |
| --- | --- | --- | --- |
| 2001  2002  2003  2004  2005  2006  2007  2008  2009  2010  2011  2012  2013  2014  2015  Total | 82  83  92  105  109  119  121  129  140  142  145  137  134  151  164  1853 | 66  373  901  1476  1894  2400  3058  3594  3946  4461  5008  5203  5574  5709  6123  49786 | 0.80  4.49  9.79  14.06  17.38  20.17  25.27  27.86  28.19  31.42  34.54  37.98  41.60  37.81  37.34  26.87 |
