# Supplementary material for: A bibliometric analysis in gene research of myocardial infarction from 2001 to 2015
Source: PeerJ. 2018 Feb 12;6:e4354. doi: 10.7717/peerj.4354 (PMC5813587; doi:10.7717/peerj.4354)
Supplement: Table S3 [file peerj-06-4354-s003.docx]

**Supplementary Table 3. The top active 10 journals that published articles on the gene research of myocardial infarction indexed in the Web of Science during 2001–2015**

| Rank | Journal | Counts(%) | IF2015 |
| --- | --- | --- | --- |
| 1  2  3  4  5  6  7  8  9  10 | CIRCULATION  ATHEROSCLEROSIS  PLOS ONE  AMERICAN JOURNAL OF PHYSIOLOGY HEART AND CIRCULATORY PHYSIOLOGY  JOURNAL OF MOLECULAR AND CELLULAR CARDIOLOGY  CARDIOVASCULAR RESEARCH  INTERNATIONAL JOURNAL OF CARDIOLOGY  JOURNAL OF THE AMERICAN COLLEGE OF CARDIOLOGY  CIRCULATION JOURNAL  CIRCULATION RESEARCH | 67(3.616)  60(3.238)  58(3.13)  48(2.59)  42(2.267)  42(2.267)  36(1.943)  32(1.727)  30(1.619)  29(1.565) | 17.047  3.942  3.057  3.324  4.874  5.465  4.638  17.759  4.124  11.551 |
