# Supplementary material for: A bibliometric analysis in gene research of myocardial infarction from 2001 to 2015
Source: PeerJ. 2018 Feb 12;6:e4354. doi: 10.7717/peerj.4354 (PMC5813587; doi:10.7717/peerj.4354)
Supplement: Table S4 [file peerj-06-4354-s004.docx]

**Supplementary Table 4 The top 10 most frequently appearing research areas that published articles** **on the gene research of myocardial infarction indexed in the Web of Science during 2001-2015**

| Rank | Research Area | Counts(%) |
| --- | --- | --- |
| 1  2  3  4  5  6  7  8  9  10 | CARDIOVASCULAR SYSTEM CARDIOLOGY  RESEARCH EXPERIMENTAL MEDICINE  GENETICS HEREDITY  CELL BIOLOGY  HEMATOLOGY  BIOCHEMISTRY MOLECULAR BIOLOGY  PHARMACOLOGY PHARMACY  PHYSIOLOGY  GENERAL INTERNAL MEDICINE  SCIENCE TECHNOLOGY OTHER TOPICS | 839(45.278)  211(11.387)  192(10.362)  176(9.498)  164(8.851)  162(8.743)  114(6.152)  106(5.72)  97(5.235)  84(4.533) |
