# Supplementary material for: A bibliometric analysis in gene research of myocardial infarction from 2001 to 2015
Source: PeerJ. 2018 Feb 12;6:e4354. doi: 10.7717/peerj.4354 (PMC5813587; doi:10.7717/peerj.4354)
Supplement: Table S5 [file peerj-06-4354-s005.docx]

**Supplementary Table 5 The top 10 cited references (CR), cited authors (CA), and active authors in the gene research of myocardial infarction indexed in the Web of Science during 2001–2015**

| Rank | Authors | Counts | Cited Author | Counts | Cited References |
| --- | --- | --- | --- | --- | --- |
| 1  2  3  4  5  6  7  8  9  10 | SCHUNKERT H  PSATY BM  HAMSTEN A  RIDKER PM  HENGSTENBERG C  ERDMANN J  ROSENDAAL FR  ZHANG L  ZEE RYL  YAMADA Y | 25  25  25  23  23  23  20  18  18  18 | Pfeffer MA  Ridker PM  Frangogiannis NG  Libby P  Ross R  Yamada Y  Samani NJ  Helgadottir A  Kathiresan S  Gardemann A | 179  143  125  119  96  96  91  84  76  62 | Samani NJ (2007)  Yamada Y (2002)  Ross R (1999)  Helgadottir A (2007)  Ozaki K (2002)  Mcpherson R (2007)  Kathiresan S (2009)  Frangogiannis NG (2002)  Broeckel U (2002)  Helgadottir A (2004) |
