# Supplementary material for: A bibliometric analysis in gene research of myocardial infarction from 2001 to 2015
Source: PeerJ. 2018 Feb 12;6:e4354. doi: 10.7717/peerj.4354 (PMC5813587; doi:10.7717/peerj.4354)
Supplement: Table S6 [file peerj-06-4354-s006.docx]

**Supplementary Table 6 the top 20 keywords in publications.**

| Rank | Keyword | Frequency |
| --- | --- | --- |
| 1  2  3  4  5  6  7  8  9  10 | myocardial infarction  coronary artery disease  heart failure  expression  polymorphism  atherosclerosis  gene expression  risk  gene  association | 829  392  309  308  299  263  234  219  208  205 |
