# Supplementary material for: A bibliometric analysis in gene research of myocardial infarction from 2001 to 2015
Source: PeerJ. 2018 Feb 12;6:e4354. doi: 10.7717/peerj.4354 (PMC5813587; doi:10.7717/peerj.4354)
Supplement: Table S7 [file peerj-06-4354-s007.docx]

**Supplementary Table 7 Top 10 Largest clusters of co-cited references among the 71 clusters**

| Cluster | Size | Silhouette | mean(Year) | Label (TFIDF) |
| --- | --- | --- | --- | --- |
| 0  1  2  3  4  5  6  7  8  9 | 62  60  54  49  36  34  32  31  23  11 | 0.902  0.822  0.891  0.889  0.806  0.943  0.874  0.892  0.921  0.939 | 2004  2007  2009  1999  2003  2009  1999  1997  1997  2004 | basic-fibroblast growth factor \| gene expression profiles  systems biology \| cell survival  chemokine \| atherogenesis  progenitor cell \| magnetic resonance imaging  inflammation \| young women  cardiac repair  extent \| cd14  density \| extent  impaired fibrinolysis \| 5g polymorphism  atherosclerosis \| inflammation-related genes |
